# Post-Anesthesia Cognitive Dysfunction in Mice Is Associated with an Age-Related Increase in Neuronal Intracellular [Ca^2+^]—Neuroprotective Effect of Reducing Intracellular [Ca^2+^]: In Vivo and In Vitro Studies

**DOI:** 10.3390/cells13030264

**Published:** 2024-01-31

**Authors:** Arkady Uryash, Alfredo Mijares, Carlos E. Lopez, Jose A. Adams, Paul D. Allen, Jose R. Lopez

**Affiliations:** 1 Division of Neonatology, Mount Sinai Medical Center, Miami, FL 33140, USA; auryash@msmc.com (A.U.); jose.adams@msmc.com (J.A.A.); 2 Centro de Biofísica y Bioquímica, Instituto Venezolano de Investigaciones Científicas, Caracas 1020, Venezuela; mijaresa@gmail.com; 3 Department of Physiotherapy, Wellmax, Miami, FL 33314, USA; carlos.lopez@deliverynetworkllc.com; 4 Leeds Institute of Biomedical & Clinical Sciences, University of Leeds, Leeds LS9 7TF, UK; paul_allen@hms.harvard.edu; 5 Department of Research, Mount Sinai Medical Center, Miami Beach, FL 33140, USA

**Keywords:** calcium, isoflurane, aging, dantrolene, cognitive deficiency, calpain, reactive oxygen species

## Abstract

***Background:*** Postoperative cognitive dysfunction (POCD) is a common disorder after general anesthesia in elderly patients, the precise mechanisms of which remain unclear. ***Methods:*** We investigated the effect of isoflurane with or without dantrolene pretreatment on intracellular calcium concentration ([Ca^2+^]_i_), reactive oxygen species (ROS) production, cellular lactate dehydrogenase (LDH) leak, calpain activity, and cognitive function using the Morris water maze test of young (3 months), middle-aged (12–13 months), and aged (24–25 months) C57BL6/J mice. ***Results:*** Aged cortical and hippocampal neurons showed chronically elevated [Ca^2+^]_i_ compared to young neurons. Furthermore, aged hippocampal neurons exhibited higher ROS production, increased LDH leak, and elevated calpain activity. Exposure to isoflurane exacerbated these markers in aged neurons, contributing to increased cognitive deficits in aged mice. Dantrolene pretreatment reduced [Ca^2+^]_i_ for all age groups and prevented or significantly mitigated the effects of isoflurane on [Ca^2+^]_i_, ROS production, LDH leak, and calpain activity in aged neurons. Dantrolene also normalized or improved age-associated cognitive deficits and mitigated the cognitive deficits caused by isoflurane. ***Conclusions:*** These findings suggest that isoflurane-induced cytotoxicity and cognitive decline in aging are linked to disruptions in neuronal intracellular processes, highlighting the reduction of [Ca^2+^]_i_ as a potential therapeutic intervention.

## 1. Introduction

Postoperative cognitive dysfunction (POCD) has been observed in elderly patients (≥65 years) who underwent general anesthesia without having any preexisting cognitive impairment [1,2,3]. Additional risk factors such as the duration and complexity of the surgical procedure, the preexistence of neurodegenerative diseases, a history of alcohol abuse, diabetes mellitus, hypertension, and atherosclerosis, as well as anesthesia, may also play a significant role in the development and intensity of POCD [4,5,6,7,8,9].

Inhalation halogenated anesthetic used in medical practice is associated with cognitive impairments in both rodents and elderly patients when administered at clinically relevant concentrations [1,8,9,10,11,12,13]. A growing body of evidence originating from various studies conducted on cell cultures and animals suggests that isoflurane has the potential to induce cellular damage. This damage has been observed in various cell types, including hippocampal slices [14], neuroglioma cells [15], and primary cortical cells [16]. It is also known that isoflurane has a detrimental effect on cell viability, triggering caspase activation and apoptosis [17], activating NFκB and increasing levels of pro-inflammatory cytokines such as TNFα, interleukin 6, and interleukin-1β [18,19]. Furthermore, isoflurane affects synaptic function by interacting with Na^+^, K^+^, and Ca^2+^ channels, which are associated with nicotinic, GABA, and glutamate neurotransmitter receptors [20]. Although the exact mechanisms underlying the neurotoxicity induced by isoflurane remain incompletely understood, studies have suggested the possibility that disruption of intracellular Ca^2+^ homeostasis is at least one of the contributing factors to cell injury caused by this anesthetic [16,21,22,23]. Furthermore, previous research has indicated that inhalation of isoflurane can contribute to neuronal damage by increasing intracellular ROS production, amplifying neuronal inflammation, and decreasing cerebral blood flow [21,22,24,25].

In a prior investigation on wild-type mice, we demonstrated a notable increase in neuronal [Ca^2+^]_i_ as the mice aged, which strongly correlated with an age-related decline in cognitive function assessed using the Morris water maze task [26]. This progressive increase in [Ca^2+^]_i_ in both cortical and hippocampal neurons appeared to be associated with an increased leak of Ca^2+^ from the sarco-endoplasmic reticulum via ryanodine receptors (RyR), as well as an augmented Ca^2+^ influx mediated by transient receptor potential canonical (TRPC) channels on the cell surface membrane [26]. Notably, our study also demonstrated that the administration of dantrolene led to a reduction in neuronal [Ca^2+^]_i_, which was associated with a decrease in calpain activity, enhancement in neuronal cell viability, and improvement in cognitive deficits in aged mice [26].

Our current investigation reinforces our previous findings and extends our understanding of the relationship between aging and [Ca^2+^]_i_ in cortical neurons in vivo and hippocampal pyramidal neurons in vitro [26]. We have also unveiled in vivo and in vitro that exposure to isoflurane intensified age-related elevations of neuronal [Ca^2+^]_i_ and neuronal ROS production, calpain activity, and LDH leakage, and it exacerbated basal age-related cognitive impairment. Most importantly, our study provides compelling evidence of the cytoprotective properties of dantrolene in aging neurons exposed to isoflurane. This discovery strongly supports the notion of neuronal Ca^2+^ dysregulation as a causal mechanism and highlights its therapeutic potential as a target to prevent POCD. Our findings suggest that modulating intracellular neuronal Ca^2+^ homeostasis using agents like dantrolene could be vital to preventing or mitigating cognitive decline associated with aging and anesthesia exposure.

## 2. Materials and Methods

### 2.1. Experimental Model

Male and female WT (C57BL6/J) mice obtained from Jackson Laboratory in Bar Harbor, ME, USA, were used as experimental animals in this study. Mice were divided into three age groups: 3 months (young), 12–13 months (middle-aged), and 24–25 months (aged). The selection of these age groups was intended to represent approximate human ages of 10, 40, and 80 years, respectively, based on the conversion ratio of a human year equivalent to nine mouse days [27]. They were provided ad libitum access to food and water and maintained under a 12-h light/dark cycle. All procedures used in this study adhered to the guidelines outlined in the National Institutes of Health Guide for the Care and Use of Laboratory Animals and the ARRIVE guidelines and were approved by the Institutional Animal Care and Use Committee of Mount Sinai Research Institute.

### 2.2. Anesthesia, Surgical Procedure, and In Vivo Measurements of Intracellular Ca^2+^ Concentration

Before the experimental day, the mice fasted overnight. Anesthesia was induced by intraperitoneal injection (ip) of ketamine (100 mg/kg) and xylazine at 5 mg/kg. Subsequently, all animals were placed on a Peltier temperature controller (WPI, Sarasota, FL, USA) to maintain their body temperature at a stable 37 °C. Their heads were meticulously positioned within a stereotaxic frame and securely immobilized with the aid of ear bars. To confirm the complete state of anesthesia of the animals, tail or toe pinches were used.

As required, anesthesia was supplemented with 5 mg ketamine/0.25 mg xylazine ip. Dexamethasone (0.2 mg/kg subcutaneous) was administered to prevent brain swelling. After shaving and disinfection of the skin above the frontal-parietal temporal lobes, a mixture of lidocaine and epinephrine was skillfully injected into the periosteum to minimize bleeding and alleviate pain. Using a pneumatic dental drill, a circular area of 1 cm diameter was meticulously marked, and the skull bone was gently displaced toward the center of the marked circle with the aid of small forceps. Petrolatum was applied around the craniotomy site to establish a “virtual chamber”, which was continuously perfused with warm sterile artificial cerebrospinal fluid [26].

Vital signs, including respiratory rate, heart rate, pulse oximetry, and rectal temperature, were continuously monitored (Kent Scientific, Torrington, CT, USA). For baseline measurements of [Ca^2+^]_i_, the anesthetized mice inhaled ambient air (Airgas, Miami, FL, USA) via a nasal mask. Subsequently, 1.5% isoflurane (1 MAC) was administered for 2 h using a small rodent anesthesia vaporizer (Kent Scientific, Torrington, CT, USA). Following the isoflurane inhalation period, we reassessed neuronal [Ca^2+^]_i_.

### 2.3. Ca^2+^-Selective Microelectrodes

Submicron double-barreled Ca^2+^ selective microelectrodes were prepared and calibrated as previously described [28]. Each Ca^2+^ microelectrode underwent individual calibration (before and after measurements) in a series of calibration solutions of known Ca^2+^ concentration (37 °C), following previously established methods [29]. This process involved the generation of calibration curves by graphing the potential recorded by the Ca^2+^ electrode against the pCa^2+^ (−log10 [Ca^2+^]) of the calibration solution. Only those Ca^2+^ microelectrodes that exhibited a Nernstian response within the range of pCa^2+^ 3 to pCa^2+^ 7 (a response of 30.5 mV per unit changes in pCa^2+^ at 37 °C) were used experimentally. As detailed previously, all measurements with a specific electrode were excluded from the analysis if a discrepancy greater than 3 mV was observed between the before and after calibration curves within the pCa^2+^ 3 and 7 [28]. After cell impalement, the resting membrane potential (V_m_) was electronically subtracted from the potential recorded by the Ca^2+^ selective Ca^2+^ barrel (V_Ca_). This subtraction led to the derivation of the Ca^2+^-specific potential signal (V_CaE_), which was then plotted onto the previously constructed calibration curve, which allowed for converting the voltage signal into the corresponding [Ca^2+^]_i_.

### 2.4. Preparation of Primary Dissociated Hippocampal Neurons

Pyramidal hippocampal neurons were obtained from male and female mice using an adapted and modified method as previously outlined [30]. In brief, after inducing anesthesia with an intraperitoneal injection of ketamine (100 mg/kg) and xylazine (5 mg/kg), young, middle-aged, and aged mice were euthanized through decapitation. After skull dissection, the brain was carefully extracted, and the hippocampus was isolated from the surrounding tissue. The isolated hippocampus was placed in ice-cold Hibernate A medium (BrainBits, Springfield, IL, USA) supplemented with B27 and glutamine (Invitrogen, Waltham, MA, USA). The hippocampal tissue was finely diced into small fragments, and the cells underwent enzymatic dissociation in a Hibernate A/B27 solution containing 2 mg/mL Papain (Worthington Biochemical Corp, Lakewood, NJ, USA). The digested tissue was transferred to a papain-free medium and triturated 15 times using a fire-polished Pasteur pipette. The triturated tissue was gently layered on the surface of an OptiPrep 1.32 gradient (Sigma-Aldrich, St. Louis, MO, USA), followed by centrifugation at 800× *g* for 15 min. Subsequently, debris above the 4 mL mark was carefully discarded, and the fraction containing neurons was collected and diluted with Hibernate A/B27 supplemented with 0.5 mM glutamine. After centrifugation at 200× *g* for 3 min, the resulting pellet was gently resuspended in Neurobasal A medium with 0.5 mM glutamine, 10 ng/mL fibroblast growth factor-2, 10 ng/mL brain-derived neurotrophic factor, 1% penicillin, and 1% streptomycin. The cells were plated on 6- or 96-well plates coated with poly-D-lysine and laminin (Falcon, Corning incorporated, Tewksbury, MA, USA) and incubated for 2 h at 37 °C, 5% CO_2_ and 10% O_2_. Subsequently, unattached cells were aspirated, and the media was replaced with Neurobasal A supplemented with 1% penicillin, 1% streptomycin, 1% glutamine, 5 ng/mL basic fibroblast growth factor, and 3 mM/L L-carnitine. The media was exchanged (50%) every 2 days. All experiments were carried out on cultured cells incubated for 6 days at 37 °C, 5% CO_2_, and 95% air.

### 2.5. Recording of Resting Membrane Potential and [Ca^2+^] In Vivo and In Vitro

In vivo recording: the resting membrane potential (RMP) and Ca^2+^ potential were recorded from the frontal-parietal cortex at a depth of <1.5 mm from the surface (the external part of the cortex) under “blind conditions” [26]. Two distinct cell populations were found based on RMP and membrane resistance values: (i) those with an average RMP of −73 ± 4 mV (*n* = 125), overshooting action potentials, and high membrane input resistance 37 ± 2 ΩM (*n* = 33), which were considered neurons [31,32]. (ii) A second population consisted of non-excitable cells with an average RMP of −56 ± 3 mV (*n* = 114) and a lower membrane resistance of 4 ± 0.5 ΩM (*n* = 41), which were considered glial cells [33,34]. We exclusively considered intracellular Ca^2+^ concentrations recorded from neurons that did not show spontaneous firing during recording. Data were selectively retained only from neurons that remained quiescent for the entire recording period (≥60 s).

In vitro recording: hippocampal neurons were impaled with the aid of an inverted microscope Axiovert 200 (Zeiss, White Plains, NY, USA). The RMP and the Ca^2+^ potentials were recorded from dissociated polarized hippocampal neurons (membrane potential ≥ −65 mV) immersed in Ringer-Locke solution supplemented with 1.5 µM tetrodotoxin (Abcam, MA, USA) [26,35]. The RMP and the Ca^2+^ potentials were recorded through a DUO 773 high-impedance amplifier (WPI, Sarasota, FL, USA) [36]. All experiments were conducted at a temperature of 37 °C.

### 2.6. Exposure to Isoflurane In Vitro

Cultured neurons were exposed to isoflurane 1.5% in a mixture of CO_2_ (5%)/air (95%) delivered from a calibrated anesthesia vaporizer (Kent Scientific, Torrington, CT, USA) in a gastight chamber inside the tissue culture incubator (Bellco Glass, Vineland, NJ, USA) for 2 h, at 37 °C.

### 2.7. Measurement of Reactive Oxygen Species

ROS production rate in isolated pyramidal hippocampal neurons was measured using the non-fluorescent cell-permeable probe 2′,7′-Dichlorofluorescin diacetate (Sigma-Aldrich, MO, USA). Neurons were loaded on day 6 of culture with 10 µM of 2′,7′-Dichlorofluorescin diacetate for 30 min at 37 °C followed by 15 min of washout with Ringer-Locke solution. The plates with cultured neurons were then placed on an inverted fluorescence microscope stage Axiovert 200 (Zeiss, White Plains, NY, USA), and the fluorescence rate (480 nm/535 nm excitation/emission) was recorded for 80–90 s. The change in the rate of the fluorescent signal rather than the intensity of the fluorescent signal was used as an index of ROS production because 2′,7′-dichlorofluorescein (DCF), the oxidized fluorescent product, is membrane-permeable and can leak out of cells over time [37]. The changed DCF intracellular concentration could introduce artifacts in the interpretation of fluorescence signals, preventing the accurate assessment of intracellular oxidation. Therefore, the rate of ROS formation was used as a metric, given its independence from the intracellular concentration of DCF.

Experiments were carried out under the following conditions: (i) untreated controls (ROS determination on day 6 of culture); (ii) exposure to 1.5% isoflurane for 2 h on day 5 of culture, and ROS determination was carried out on day 6 of culture); (iii) after incubation with dantrolene (20 µM) for 48 h (beginning day 4 and ROS determination on day 6 of culture); (iv) after incubation with dantrolene (20 µM) for 48 h (beginning day 3 of culture), exposed to 1.5% isoflurane for 2 h in the presence of dantrolene on day 5 of culture (ROS determination on day 6 of culture). The slope of dichlorofluorescein fluorescence for all groups was normalized to the average rate recorded from young untreated control neurons.

### 2.8. Measurements of Calpain Activity

The activity of calpain I and II in hippocampal neurons was determined using the calpain-glo protease assay (Promega, Madison, WI, USA) under the same four experimental conditions as ROS measurements. Young, middle-aged, and aged neurons were incubated with the calpain-glo reagent at 37 °C on the sixth day of culture for 10 min. After incubation, luminescence readings were obtained using a microplate reader (Molecular Devices, Boston, MA, USA). Luminescence values are reported normalized to values from young untreated neurons.

### 2.9. Lactate Dehydrogenase Assay

The lactate dehydrogenase leak was assessed over 24 h using a colorimetric assay kit (Abcam, Woburn, MA, USA). This assay measures the LDH activity that leaks from the cytosol of damaged neurons into the supernatant [38]. The absorbance at 450 nm was measured using a microplate reader (Molecular Devices, Boston, MA, USA). The 24-h LDH leak from hippocampal neurons was evaluated on cultured cells under the same four experimental conditions as ROS measurements (beginning day 5 and determining LDH on day 6 of culture). The LDH leak values were normalized to the average leak obtained from young, untreated neurons.

### 2.10. Morris Water Maze Test

The Morris water maze test assessed the cognitive function of mice, specifically spatial learning and memory [39]. Briefly, young, middle-aged, and aged mice received 3 trials per day for 4 consecutive training days (typically Monday–Thursday) (training period). Mice were allowed to swim (water temperature 26 ± 1.5 °C for a maximum of 60 s); if a mouse could not locate the platform within 60 s, it was gently directed to the platform, rescued, and towel dried. The mice were left on the platform for 10–20 s. After each trial, each mouse was towel-dried and kept warm before returning to its regular cage. The platform was removed on the fifth day (probe trial) and measured: (a) the escape latency time (the time taken by the mice to reach the platform), (b) the swim speed, (c) the path length (the accumulated distance traveled to reach the platform), (d) the time spent by the mice in the target quadrant that contained the platform, and (e) the number of times the mouse crossed over the area where the platform was previously hidden. The behavior of the mice in the pool was recorded using a video tracking system (WatermazeScan, Reston, VA, USA), and stored the same test was run. The following parameters are for later analysis.

**Figure 1:**
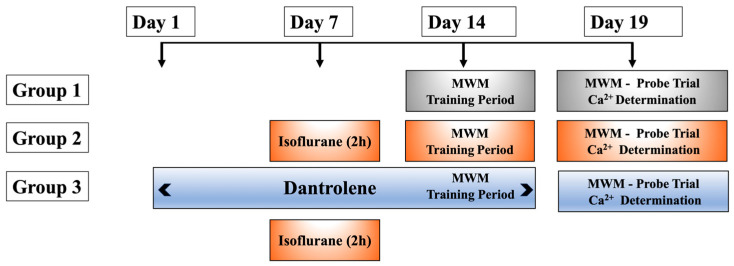
Timely diagram of experimental procedures to assess cognitive function using the Morris water maze (MWM). Young, middle-aged, and aged mice were randomly assigned into three groups (*n* = 8 mice per age group and experimental condition). Group 1: control group; Group 2: the effect of isoflurane (1.5%) was examined on cognitive functions; Group 3: the effect of dantrolene (1 mg/kg ip) on isoflurane-induced changes in cognitive performance was determined. [Ca^2+^]_i_. was measured in vivo using Ca^2+^ selective microelectrodes at the end of the protocol (day 19).

Young, middle-aged, and aged mice were randomly divided into 3 groups (*n* = 8 mice per age group and experimental condition) (Figure 1): Group 1: Control group. Mice did not receive any treatment prior to training, during the training period (4 days), and for the probe trial (day 5); Group 2: Effect of isoflurane on cognitive performance. Mice were exposed to 1.5% isoflurane by nasal mask for 2 h on day 7; on day 14, they began the training period (for 4 days), and the probe trial was carried out on day 19 [40,41]. The concentration of 1.5% isoflurane was chosen because it represents approximately 1 MAC [42] and is similar to the concentrations used in clinical practice. Group 3: Effect of dantrolene on isoflurane-induced changes in cognitive performance and [Ca^2+^]_i_. All mice in this group received dantrolene (1 mg/kg, i.p. day) for 7 days, then exposed to isoflurane for 2 h on day 7, continuously with dantrolene until the training period of the MWM test was completed. After the MWM studies, [Ca^2+^]_i_ was measured in vivo in cortical neurons from all 3 experimental groups (Figure 1).

### 2.11. Solutions

The artificial sterile cerebrospinal fluid contained (in mM) 135 NaCl, 1.8 KCl, 26 NaHCO_3_, 1.25 NaH_2_PO_4_, 2 CaCl_2_, 1 MgCl_2_, and 5 glucose (pH 7.4). Ringer-Locke solution contained (in mM): 135 NaCl, 5 KCl, 2 CaCl_2_, 1 MgCl_2_, 5 glucose, 3.6 NaHCO_3_ (pH 7.4). Artificial cerebrospinal fluid and Ringer-Locke solution were aerated with a mixture of 95% O_2_ and 5% CO_2_ for all experiments. For in vitro experiments, 20 µM dantrolene (Sigma-Aldrich, MO, USA) was prepared by adding concentrated stocks in dimethyl sulfoxide to the Ringer-Locke solution. For control experiments, a similar volume of dimethyl sulfoxide was added to the Ringer-Locke solution. For intracellular Ca^2+^ recordings in vitro, Na^+^ channel blocker tetrodotoxin (1.5 µM) (Abcam, Waltham, MA, USA) was added to the Ringer-Locke solution to prevent spontaneous depolarizations [26].

### 2.12. Statistical Analysis

All data are presented as mean ± standard deviation (SD), with *n_cells_* representing the number of neurons in which measurements were successfully conducted. We used histograms and the D’Agostino & Pearson test to assess the data distribution. Statistical analyses were performed by ANOVA with Tukey’s post-test for multiple measurements with *p* < 0.05 considered significant (GraphPad Software 10, Boston, MA, USA).

## 3. Results

### 3.1. Effect of Isoflurane on [Ca^2+^]_i_ in Cortical Neurons (In Vivo)

Earlier research has provided mounting evidence that volatile anesthetics, particularly isoflurane, can cause neuronal damage by disrupting the regulation of [Ca^2+^]_i_ [22,23].

**Figure 2:**
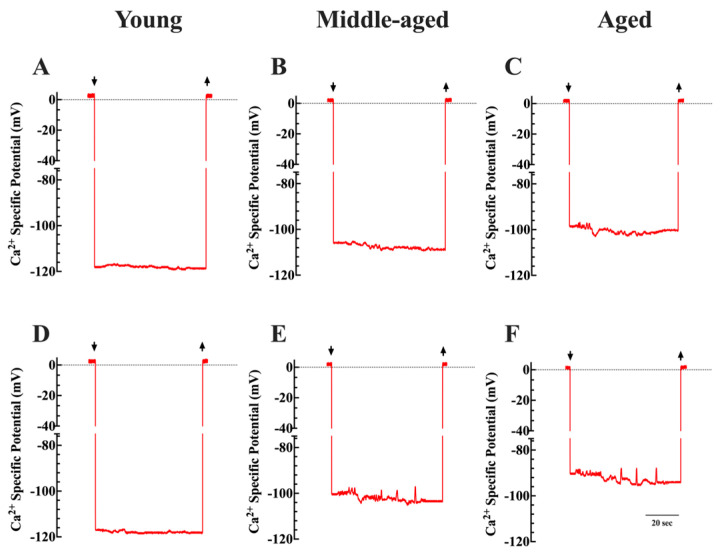
Recordings of Ca^2+^-specific potentials from young, middle-aged, and aged neurons (in vivo). (**A**–**C**) show typical traces of Ca^2+^-specific potentials recorded from neurons corresponding to the respective age categories. After proper calibration, the actual [Ca^2+^]_i_ was as follows: in the young neuron, it was 128 nM, while in the middle-aged neuron, it was 224 nM, and in the aged neuron, it was 332 nM. After exposure to isoflurane, the calibrated [Ca^2+^]_i_ were 119 nM in the young neuron (**D**), 320 nM in the middle-aged neuron (**E**), and 734 nM in the aged neuron (**F**). Top left and right arrows: Ca^2+^ microelectrode impalement and muscle withdrawing. The calibration bar represents 20 s.

**Figure 3:**
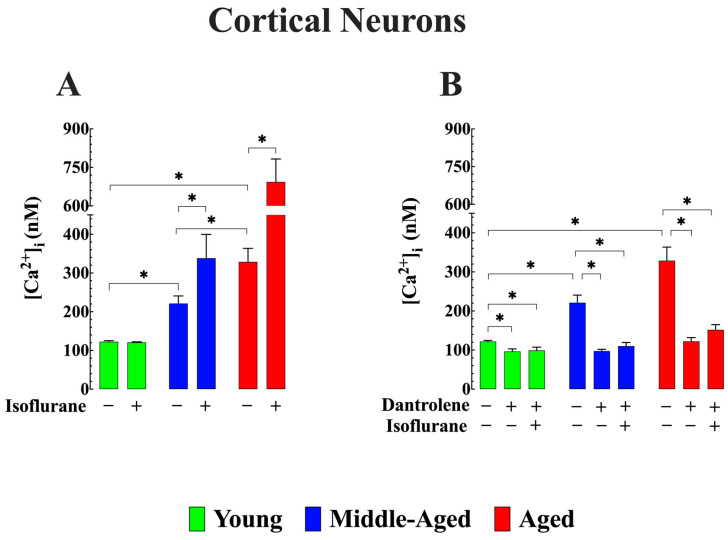
Dantrolene reduces abnormal resting intracellular [Ca^2+^] and inhibits isoflurane-induced further elevation in aged cortical neurons (in vivo). (**A**) The average [Ca^2+^]_i_ in aged neurons was more elevated than in young cortical neurons. In young mice, inhalation of 1.5% isoflurane did not cause a significant change in [Ca^2+^]_i_. However, middle-aged and aged cortical neurons significantly increased [Ca^2+^]_i_. (**B**) In a second cohort of mice, pretreatment with dantrolene decreased [Ca^2+^]_i,_ in all age groups, and isoflurane inhalation did not increase [Ca^2+^] in middle-aged mice and in aged neurons minimized the elevation of [Ca^2+^]. *n*_mice_ = 7/age group-experimental condition. Values are expressed as mean ± S.D. * denotes *p* < 0.05.

Figure 2 shows the recording of Ca^2+^ potentials in vivo from young, middle-aged, and aged neurons. The average [Ca^2+^]_i_ was 123 ± 3 nM (*n_cells_* = 9) in young cortical neurons, while in middle-aged neurons, it was 221 ± 20 nM (*n_cells_* = 10), and in aged cortical neurons, it was 329 ± 35 nM (*n_cells_* = 11) (*p* < 0.05 when compared young to middle-aged, and aged) (Figure 3A). These values were consistent with our previous findings on the effects of aging on the regulation of [Ca^2+^]_i_ [26]. Upon exposure to 1.5% isoflurane, middle-aged neurons showed a significant increase in [Ca^2+^]_i,_ reaching 338 ± 61 nM (*n_cells_* = 8), and in aged neurons, 694 ± 89 nM (*n_cells_* = 9) (*p* < 0.05 when compared to nonexposed age-matched neurons, in both groups) (Figure 3A). Notably, inhalation of isoflurane did not result in a noticeable alteration of [Ca^2+^]_i_ in neurons from young mice (121 ± 2 nM, (*n_cells_* = 7, *p* < 0.05 compared to not exposed young neurons) (Figure 3A).

### 3.2. Reduction of Abnormal [Ca^2+^]_i_ Averts Additional Elevation Induced by Isoflurane in Aged Cortical Neurons (In Vivo)

In a second cohort of mice, we pretreated the three groups of mice with dantrolene (1 mg/kg/day, i.p.) for 7 days. It has previously been shown that dantrolene effectively reduces [Ca^2+^]_i_ in both skeletal muscle and neurons [26,43,44]. Consistent with our earlier findings, the administration of dantrolene resulted in a decrease in [Ca^2+^]_i_. Specifically, in young cortical neurons, [Ca^2+^]_i_ was reduced from 122 ± 3 nM (*n_cells_* = 9) to 97 ± 6 nM (*n_cells_* = 7) in middle-aged neurons from 221 ± 20 nM (*n_cells_* = 10) to 98 ± 4 nM (*n_cells_* = 7) and in aged neurons from 329 ± 35 nM (*n_cells_* = 11) to 122 ± 10 nM (*n_cells_* = 9) (*p* < 0.05 compared to age-matched untreated neurons) (Figure 3B). Dantrolene pretreatment successfully blocked the isoflurane-induced elevation of [Ca^2+^]_i_ in middle-aged neurons and significantly mitigated the increase observed in aged cortical neurons (Figure 3B).

### 3.3. Cytoprotective Effects of Lowering [Ca^2+^]_i_ on Isoflurane-Induced Elevation of [Ca^2+^]_i_ in Aged Hippocampal Neurons (In Vitro)

**Figure 4:**
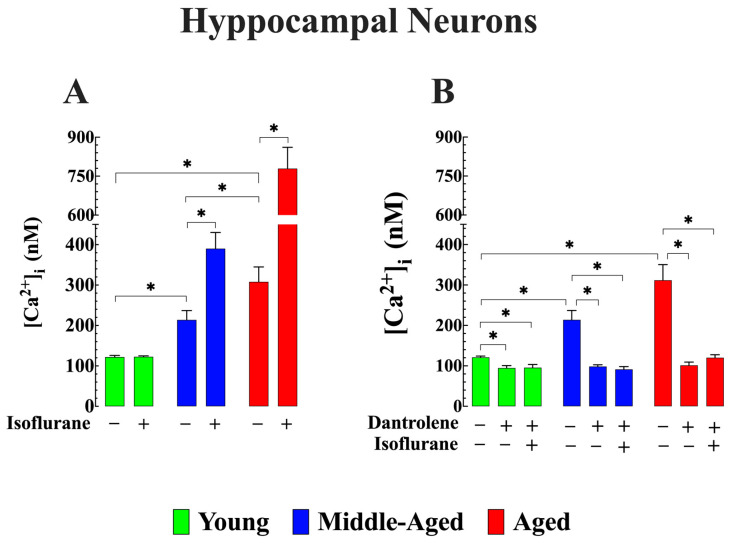
Dantrolene reduces aberrant resting [Ca^2+^]_i_ and prevents isoflurane-induced intracellular Ca^2+^ overload in aged hippocampal neurons (in vitro). (**A**) The average [Ca^2+^]_i_ was more elevated in aged neurons than young hippocampal neurons. Isoflurane (1.5%) did not cause an increase [Ca^2+^]_i_ in young neurons; however, in middle-aged and aged neurons, [Ca^2+^] was further elevated. (**B**) Pretreatment with dantrolene reduced [Ca^2+^]_i_ in all neurons and blocked the isoflurane-induced increase in [Ca^2+^]_i_ in middle-aged and diminished the magnitude of the elevation in aged neurons. *n*_mice_ = 7/age group-experimental condition. Values are expressed as mean ± S.D. * denotes *p* < 0.05.

The hippocampus plays an important role in learning and memory formation [7]. Previous research has indicated that isoflurane can alter neurons’ functions in the hippocampus [45]. We investigated the effect of 2 h of exposure to isoflurane on [Ca^2+^]_i_ in pyramidal hippocampus neurons obtained from young, middle-aged, and aged mice. Remarkably, the patterns observed in hippocampal neurons mirrored the age-elevation of [Ca^2+^]_i_ found in vivo in cortical neurons across the three age groups(122 ± 4 nM (*n_cells_* = 14) in young, 213 ± 23 nM (*n_cells_* = 18) n middle-aged and 308 ± 37 nM (*n_cells_* = 9) in aged neurons; *p* < 0.05 compared to young neurons). Isoflurane caused elevation in [Ca^2+^]_i_ in middle-aged 390 ± 40 nM (*n_cells_* = 11) and aged neurons 779 ± 82 nM (*n_cells_* = 10) (*p* < 0.05 compared to untreated neurons of the same age), without effect on young neurons (123 ± 2 nM, *n_cells_* = 12) (Figure 4A). Likewise, when hippocampal neurons were pretreated with dantrolene (20 µM) for 48 h, the resulting decrease in [Ca^2+^]_i_ closely resembled the reduction observed in vivo after dantrolene pretreatment and the prevention of intracellular Ca^2+^ overload caused by isoflurane (*p* > 0.05) (Figure 4B). A comparable reduction in [Ca^2+^]_i_ has been noted in aging neurons when subjected to incubation in a low calcium solution or pre-exposed to SAR7334 (1 µM), a transient receptor potential canonical (TRPC) channel blocker [26]. Furthermore, preincubation in SAR7334 also inhibits or reduces the magnitude of [Ca^2+^]_i_ elevation induced by isoflurane in aged neurons (Supplemental Figure S1). This observation implies that the effect of isoflurane on [Ca^2+^]_i_ in aging neurons is likely mediated through a Ca^2+^ pathway that is sensitive to SAR7334, particularly targeting TRPC3 and 6, given the pharmacological specificity of the compound [46].

### 3.4. Dantrolene Reverses the Elevated Production of ROS and Prevents Its Increase by Isoflurane in Aged Neurons

**Figure 5:**
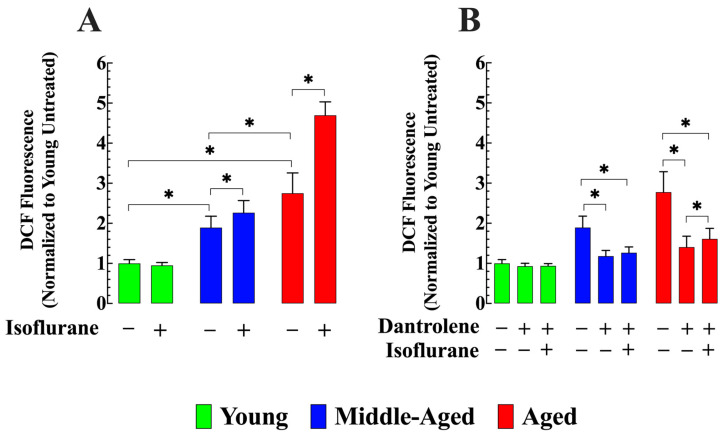
Dantrolene decreases abnormal ROS production and prevents isoflurane from causing additional elevation in aged hippocampal neurons. (**A**) ROS production in middle-aged and aged hippocampal neurons was significantly higher than in young neurons. Isoflurane further increased ROS production in middle-aged and aged neurons, with no significant increase in young neurons. (**B**) pretreatment with dantrolene (20 µM for 48 h) significantly decreased ROS production in middle-aged and aged neurons and prevented or mitigated the increase induced by isoflurane. Dantrolene did not significantly affect young neurons. *n*_mice_ = 5/age group-experimental condition. Values are expressed as mean ± S.D. * denotes *p* < 0.05.

The accumulation of ROS induced by isoflurane exposure has been implicated in the opening of the mitochondrial permeability transition pore, leading to neuronal cell death [47]. We used 2′,7′-dichlorofluorescein to assess ROS formation as a fluorescent indicator. Comparative analysis revealed that middle-aged hippocampal neurons exhibited 89% (*n_cells_* = 17) higher ROS formation, and aged neurons showed an increase of 175% (*n_cell_* =19) compared to young neurons (*p* < 0.05 compared to young neurons). After exposure to isoflurane, a further elevation in ROS production from baseline level was observed in middle-aged (20%%, *n_cells_* = 17) and aged neurons 71% (*n_cells_* = 15, *p* < 0.05 compared to untreated neurons of the same age). No effect was observed in young neurons (*p* > 0.05 compared to untreated young neurons) (Figure 5A). Interestingly, pretreatment with dantrolene reduced ROS formation by 38% (*n_cells_* = 15) in middle-aged neurons and 49% (*n_cells_* = 15) in aged neurons (*p* < 0.05 compared to untreated age-matched neurons) (Figure 5B). No significant effects were observed in young neurons (*n_cells_* = 13, *p* > 0.05 compared to untreated young neurons). Furthermore, pretreatment with dantrolene prevented the increase in ROS formation associated with exposure to isoflurane in middle-aged neurons (*n_cells_* = 13, *p* > 0.05 compared to middle-aged neurons untreated with dantrolene) and significantly attenuated it in aged neurons (*n_cells_* = 13, *p* < 0.05 compared to dantrolene untreated age neurons) (Figure 5A,B).

### 3.5. Dantrolene Diminish Calpain Activity and Prevents Isoflurane-Induced Elevation in Older Neurons

**Figure 6:**
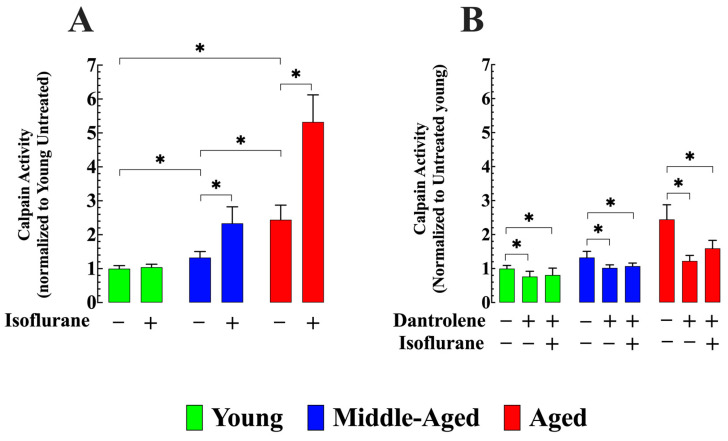
Dantrolene reduces elevated basal calpain activity and prevents isoflurane-induced additional elevation in aged hippocampal neurons. (**A**) Calpain activity was significantly elevated in middle-aged and aged neurons compared to young hippocampal neurons. Isoflurane (1.5%) did not cause a change in calpain activity in young neurons; however, it was significantly increased in middle-aged and aged neurons. (**B**) Pretreatment with dantrolene (20 µM) for 48 h reduced calpain activity in middle-aged and aged neurons, blocked the isoflurane-induced increase in calpain activity in middle-aged neurons, and attenuated elevation in aged neurons. *n*_mice_ = 4/age group-experimental condition. Values are expressed as mean ± S.D. * denotes *p* < 0.05.

An increase in [Ca^2+^]_i_ is believed to initiate a series of biochemical events, including the activation of calpain, which can ultimately result in neuronal damage [48]. Compared to young hippocampal neurons, middle-aged neurons showed a 33% increase in calpain activity (*n_cells_* = 19, *p* < 0.05 compared to young neurons), while aged neurons exhibited a 144% increase (*n_cells_* = 20, *p* < 0.05 compared to young neurons) (Figure 6A). After a two-hour exposure to isoflurane, the elevated resting calpain activity further increased by 76% in middle-aged neurons (*n_cells_* = 16, *p* < 0.05 compared to untreated middle-aged neurons) and 118% in aged neurons (*n_cells_* = 17, *p* < 0.05 compared to untreated aged neurons). No significant effect was observed in young hippocampal neurons (*n_cells_* = 19, *p* > 0.05) (Figure 6A). However, when treated with dantrolene, the calpain activity decreased by 23% in middle-aged neurons (*n_cells_* = 13, *p* < 0.05 compared to untreated middle-aged neurons) and by 50% in aged hippocampal neurons (*n_cells_* = 20, *p* < 0.05 compared to untreated aged neurons) (Figure 6B). Moreover, dantrolene inhibited the increase in calpain activity caused by exposure to isoflurane in middle-aged neurons and mitigated the extent of the increase in aged neurons (Figure 6B).

### 3.6. Dantrolene Attenuates LDH Leak and Inhibits Isoflurane-Induced Elevation in Aging Neurons

**Figure 7:**
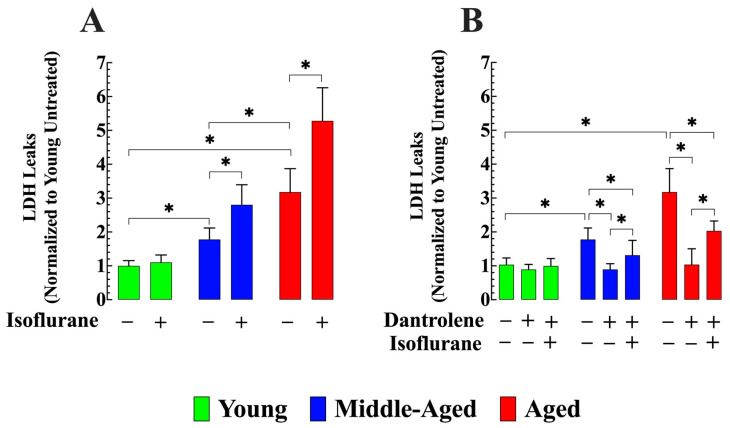
Dantrolene reduces basal LDH leak and prevents further elevation caused by isoflurane in aged hippocampal neurons. (**A**) The 24-h LDH leak was higher in middle-aged and aged neurons than in young neurons. Exposure to isoflurane increased LDH leakage in middle-aged neurons and aged neurons. No significant changes in LDH were observed in young neurons exposed to isoflurane. (**B**) Preincubation with dantrolene (20 µM) reduced LDH in middle-aged and aged neurons, prevented the isoflurane-induced increase in LDH in untreated middle-aged neurons, and attenuated it in aged neurons. *n*_mice_ = 5/age group-experimental condition. Values are expressed as mean ± S.D. * denotes *p* < 0.05.

LDH cell leakage serves as a measure of cell damage [49]. Compared to young hippocampal neurons, middle-aged neurons exhibited 77% greater LDH leak at 24 h (*n_cells_* = 15, *p* < 0.05 compared to young neurons), while aged neurons showed an increase of 217% (*n_cells_* = 15, *p* < 0.05 compared to young neurons) (Figure 7A). Exposure to isoflurane significantly increased LDH leak by 58% in middle-aged neurons (*n_cells_* = 15, *p* < 0.05 compared to untreated middle-aged neurons) and 71% in aged neurons (*n_cells_* = 17, *p* < 0.05 compared to untreated aged neurons. No effect was observed on young neurons (*n_cells_* = 12, *p* > 0.05 compared to untreated) (Figure 7A). Pretreatment with dantrolene significantly reduced LDH leak in middle-aged by 50% (*n_cells_* = 15, *p* < 0.05 compared to untreated middle-aged neurons) and aged neurons and 67% (*n_cells_* = 20, *p* < 0.05 compared to untreated aged neurons), while it exhibited no significant effect on young neurons (*n_cells_* = 16, *p* > 0.05 compared to untreated young neurons (Figure 7B). Furthermore, pretreatment with dantrolene successfully prevented the increase in LDH leak caused by exposure to isoflurane in middle-aged neurons (*n_cells_* = 16, *p* < 0.05 compared to isoflurane-treated middle-aged neurons) and significantly reduced the LDH leak in aged neurons (*n_cells_* = 15, *p* < 0.05 to isoflurane-treated aged neurons) (Figure 7B).

### 3.7. Dantrolene Enhances Cognitive Function and Prevents Isoflurane-Induced Further Decline in Aged Mice

**Figure 8:**
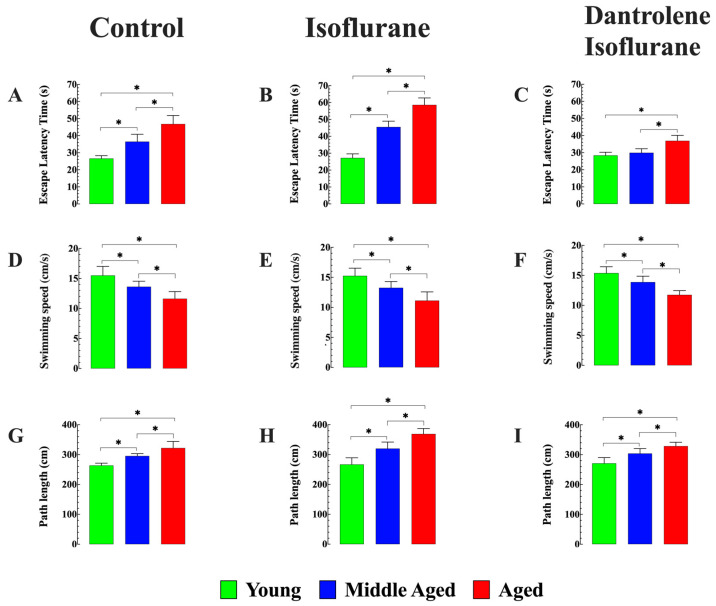
Effects of isoflurane and dantrolene on escape latency, swimming speed, and path length. (**A**–**C**) The escape latency times of middle-aged and aged mice in the probe trial (MWM day 5) were significantly different compared to young mice. Exposure to isoflurane (1.5 *v*/*v*) worsens the escape latency time in middle-aged and aged mice without effect on young mice. Dantrolene pretreatment before isoflurane inhalation prevented the effect of isoflurane on the escape latency time in middle-aged mice and partially prevented the effect of isoflurane in aged mice. (**D**–**F**) Swimming speeds decreased in middle-aged and aged mice compared to their younger counterparts (24 < 12 < 3 months). No detectable effect was observed after exposure to isoflurane inhalation or pretreatment with dantrolene and isoflurane inhalation. (**G**–**I**) The path length of middle-aged and aged mice differed significantly from that of young mice. Isoflurane aggravated the trajectories observed in middle-aged and aged mice without effect on young mice. Dantrolene improved inconsistent trajectories in middle-aged and aged mice without affecting young mice. Values are expressed as mean ± S.D; * denotes *p* < 0.05.

Our previous study showed progressive cognitive deficits in middle-aged and aged mice compared to young mice, as evidenced by their performance in the MWM [26]. We confirmed that both middle-aged (36 ± 4.3) (seconds (s), *n* = 8, *p* < 0.05 compared to young mice) and aged mice (47 ± 4.9 s, *n* = 8, *p* < 0.05 compared to young mice) exhibited significantly longer escape latency than young mice (26 ± 1.6 s) (Figure 8A). Further investigation revealed that exposure to isoflurane exacerbated escape latency in middle-aged (46 ± 3.4 s, *n* = 8, *p* < 0.05 compared to untreated middle-aged mice) and aged mice (58 ± 4.1 s, *n* = 8, *p* < 0.05 compared to untreated aged mice), while no discernible effect was observed in young mice (27 ± 2. s, *n* = 8, *p* > 0.05 compared to young untreated mice) (Figure 8B). Preventive treatment with dantrolene before inhalation of isoflurane (i) reversed abnormal escape latency in middle-aged mice (30 ± 2.3 s, *n* = 8, *p* > 0.05 compared to young mice) and fully prevented the exacerbating effects of isoflurane (*p* < 0.05 compared to isoflurane treated middle-aged mice) (Figure 8C): (ii) In aged mice, dantrolene partially reversed the abnormal age-dependent escape latency (36 ± 3.2 s, *n* = 8, *p* < 0.05 compared to dantrolene untreated aged mice) and mitigated the effect of isoflurane (*p* < 0.05 compared to isoflurane-treated aged mice). No significant effect was observed in young mice (28 ± 2 s, *n* = 8, *p* > 0.05 compared to young untreated mice).

Although escape latency is one of the classical measurements in MWM, factors not related to the cognitive abilities of the animal, such as variations in swimming speed with age, can interfere with escape latency measurements [50]. We confirmed that middle-aged (13 ± 0.46 cm/s. *n* = 8) and aged mice (11 ± 0.92 cm/s, *n* = 8) significantly swim more slowly than their younger counterparts (16 ± 1.5 cm/s, *n* = 8) (*p* < 0.05 when compared middle-aged and aged mice *versus* young mice) (Figure 8D). Importantly, neither isoflurane administration alone nor the dantrolene pretreatment (at the dose used in this study) followed by isoflurane inhalation had any significant impact on swimming speeds across different age groups (Figure 8E,F). To minimize the influence of swimming speed on our determination of escape latency, the path length was also measured, representing the total distance covered by the mouse to reach the platform in all three age groups. Young mice consistently selected more direct routes to reach the platform, resulting in shorter path lengths (265 ± 13 cm, *n* = 8); in contrast, middle-aged (298 ± 10 cm, *n* = 8) and aged mice (32 ± 22 cm, *n* = 8) exhibited more erratic trajectories, leading to longer path lengths, as illustrated in Figure 8G–I.

**Figure 9:**
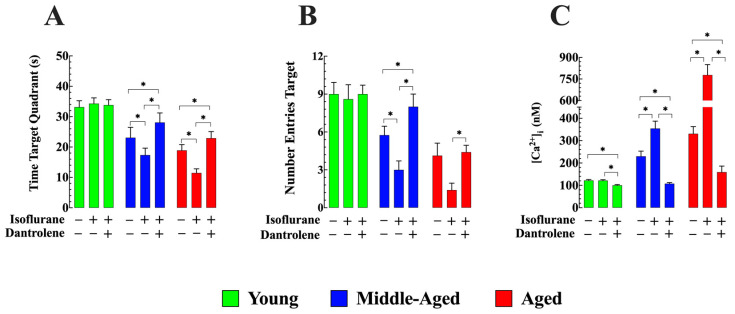
Dantrolene improves preexisting cognitive impairment and prevents isoflurane-induced further decline in aged mice. (**A**) The time spent in the target quadrant was significantly reduced in middle-aged and aged mice compared to young mice. Isoflurane further decreased the time spent in the target quadrant in middle-aged and aged mice but had no effect in young mice. Dantrolene treatment before, during, and after isoflurane treatment increased the time spent in the target quadrant in middle-aged and aged mice compared to the control and prevented the decrease associated with isoflurane alone. No significant effect on the time spent in the target quadrant was observed in young mice. (**B**) The number of times a mouse crossed the area where the platform was hidden (target area) was significantly reduced in middle-aged and aged mice compared to young mice). Isoflurane reduced the number of times mice crossed the target area in middle-aged and old mice but had no effect in young mice. Dantrolene pretreatment prevented the decrease in the number of times a mouse crossed the target area elicited by isoflurane in middle-aged and aged mice. (**C**) The determination of [Ca^2+^]_i_ in the same cohorts of mice after the MWM testing was completed showed (i) elevation of [Ca^2+^]_i_ in cortical neurons of middle-aged and aged mice compared to young mice, (ii) inhalation of isoflurane worsened [Ca^2+^]_i_ dysfunction in middle-aged and aged neurons, and (iii) pretreatment with dantrolene completely prevents the elevation of [Ca^2+^]_i_ in middle-aged neurons and significantly reduced it in aged neurons. *n*_mice_ = 8/age group-experimental condition. Values are expressed as mean ± S.D. * denotes *p* < 0.05.

Furthermore, spatial learning was assessed using the following parameters: (i) the time the mouse spent in the target quadrant and (ii) the number of times the mouse crossed the platform area. Compared to young mice (33 ± 2.1 s), both middle-aged (23 ± 3.3 s, *n* = 8) and aged mice (18 ± 1 s, *n* = 8) exhibited a mouse-reduced time spent in the target quadrant (comparison between young and middle-aged mice, as well as aged mice, was significant *p* < 0.05) (Figure 9A). Isoflurane inhalation significantly impaired memory retention, as evidenced by reduced time spent in the target quadrant, middle-aged (17 ± 2.2 s, *n* = 8), and aged mice (11 ± 1.3 s, *n* = 8, *p* < 0.05 compared to untreated middle-aged and aged mice, respectively) (Figure 9A). No effect was observed in young mice (34 ± 2.4 s, *p* > 0.05 compared to untreated young mice). Dantrolene pretreatment effectively improved memory retention deficiency in middle-aged (28 ± 3.1 s, *n* = 8, *p* < 0.05 compared to untreated middle-aged mice) and aged mice (23 ± 2.1 s, *n* = 8, *p* < 0.05 compared to untreated aged mice). Furthermore, it counteracted the negative impact of isoflurane on the time spent in the target quadrant (Figure 9A).

When examining the number of times the mouse crossed the platform area, both middle-aged (6 ± 0.5 times, *p* < 0.05 compared to young mice) and aged mice (4 ± 0.8 times, *p* < 0.05 compared to young mice) exhibited a significantly reduced frequency compared to young mice (9 ± 1 times) (Figure 9B). Interestingly, isoflurane inhalation further impaired memory retention in middle-aged and aged mice, as evidenced by fewer crossings in the platform area (3 ± 0.7 times and 1.4 ± 0.6 times, respectively) (*p* < 0.05 compared to untreated middle-aged mice and age mice). On the contrary, no discernible effect was observed in young mice (8 ± 1 times, *p* > 0.05 compared to young mice not treated (Figure 9B). Importantly, dantrolene pretreatment successfully improved preexisting cognitive deficit in middle-aged (8 ± 1 times) and aged mice (4 ± 1 times) but also counteracted the negative impact of isoflurane in aged mice, as indicated by the number of crossings in the platform area (Figure 9B).

After completion of the MWM studies, in vivo measurements of cortical neuronal [Ca^2+^]_i_ revealed that exposure to isoflurane-induced a further increase in neuronal [Ca^2+^]_i_ from 230 ± 23 nM *(n_cells_* = 9) to 354 ± 33 nM (*n_cells_* = 8) in middle-aged and in aged from 331 ± 32 nM (*n_cells_* = 10) to 778 ± 72 nM (*n_cells_* = 8). No effect was observed in young mice (122 ± 3 nM (*n_cells_* = 9) and after 121 ± 4 nM (*n_cells_* = 8). Dantrolene pretreatment reduced [Ca^2+^]_i_ in cortical neurons in all age groups and prevented elevation of [Ca^2+^]_i_ induced by isoflurane in middle-aged neurons and aged neurons (Figure 9C).

## 4. Discussion

POCD refers to disorders affecting orientation, attention, perception, consciousness, and judgment that are seen in elderly patients (65 years and older) after general anesthesia in the absence of preexisting mental disorders [1,2,3]. Although this decline usually resolves within days, weeks, or months, it significantly affects the quality of life of patients and may potentially accelerate the progression of undiagnosed Alzheimer’s disease [51]. Risk factors that increase the likelihood of POCD include advanced age, preexisting cerebral, cardiac, and vascular conditions, alcohol misuse, as well as intraoperative and postoperative complications. The exact pathophysiology and underlying mechanisms of postoperative cognitive decline remain unclear or, at best, incompletely understood [52]. However, accumulating evidence from cell culture and animal studies suggests that one potential mechanism underlying anesthetic-induced neural injury could be the dysregulation of intracellular Ca^2+^ [21,23,52]. Uncovering the mechanisms causing postoperative cognitive dysfunction is one of the most challenging problems in anesthesiology and neuroscience. To address this enigma, we combined in vivo and in vitro electrophysiological measurements, metabolic markers, cell integrity evaluations, and studies looking for cognitive deficits in various age groups, including young, middle-aged, and aged mice.

Our study yielded the following findings:

1. It reaffirmed our previous discovery that middle-aged and aged WT C57BL6/J mice exhibit chronically elevated [Ca^2+^]_i_ in cortical neurons in vivo and hippocampal pyramidal neurons in vitro compared to young mice [26].
2. In vivo and in vitro exposure to isoflurane led to a significant increase in [Ca^2+^]_i_ in middle-aged and aged cortical and hippocampal neurons but did not affect neuronal [Ca^2+^]_i_ in young mice. However, dantrolene treatment reduced the basal level of [Ca^2+^]_i_ across all age groups and prevented or reduced the elevation of [Ca^2+^]_i_ associated with exposure to isoflurane in middle-aged and aged neurons.
3. Middle-aged and aged hippocampal pyramidal neurons had higher ROS production, elevated calpain activity, and increased LDH leak compared to young neurons. These age-related changes were mitigated by dantrolene treatment. The administration of isoflurane further increased all three parameters over base levels, but pretreatment with dantrolene prevented or attenuated the effects of isoflurane.
4. The elevated [Ca^2+^]_i_ observed in middle-aged and aged animals was associated with age-related cognitive deficits evaluated using MWM. Isoflurane further aggravated cognitive deficits in aged mice. As seen with the increase of [Ca^2+^]_i_ dantrolene pretreatment prevented or alleviated the isoflurane-induced performance decline. The ability of dantrolene to prevent isoflurane-induced performance decline was associated with reduced neuronal [Ca^2+^]_i._

In young quiescent neurons, [Ca^2+^]_i_ is carefully regulated at low levels (100–120 nM) despite the presence of a significant concentration gradient with extracellular fluid [35]. The tight control of neuronal [Ca^2+^]_i_ relies on balancing Ca^2+^ influx across the plasma membrane and release from intracellular stores, coupled with intracellular sequestration and extrusion mechanisms [53]. Any disturbances in the mechanisms that regulate [Ca^2+^]_i_ can ultimately result in a sustained elevation or reduction in the neuronal [Ca^2+^]_i._ Our study reconfirmed our previous findings that there is an age-related increase in [Ca^2+^]_i_ in cortical (in vivo) and cultured hippocampal pyramidal neurons (in vitro), which appears to be mediated by ROS induced Ca^2+^ leak from the ryanodine receptor (RyR) and increased Ca^2+^ influx across the plasmalemma through transient receptor potential canonical channels (TRPC) [26]. The resulting intracellular Ca^2+^ overload can trigger events that cause cell death by activating proteases and caspase or initiating other catabolic processes facilitated by lipases and nucleases [54,55]. Dysregulation of [Ca^2+^]_i_ has also been implicated in the pathogenesis of various neurodegenerative disorders such as Alzheimer’s disease and brain aging [56,57].

More specifically related to POCD, we presented evidence indicating that exposure to isoflurane, a frequently used inhalational anesthetic, aggravated the already abnormal basal [Ca^2+^]_i_ levels already observed in aging neurons. Isoflurane activates NFκB and increases levels of pro-inflammatory cytokines such as TNFa, interleukin 6, and interleukin-1β [18,19]. Additionally, isoflurane induces sarcoendoplasmic stress by activating 1,4,5-triphosphate and RyR receptors, leading to neurotoxic damage and cognitive impairment [7,14,21,22]. Based on the present study, we hypothesize that isoflurane neurotoxicity in aged neurons is mediated by worsening preexisting alterations of intracellular [Ca^2+^] since the pharmacological reduction of neuronal [Ca^2+^]_i_ prevented or minimized the Ca^2+^ overload and neurotoxicity induced by isoflurane.

In the current study, we showed that dantrolene treatment significantly reduced [Ca^2+^]_i_ in cortical -in vivo- and hippocampal -in vitro- neurons from middle-aged and aged mice and prevented or mitigated the further elevation of [Ca^2+^]_i_ induced by isoflurane in aged neurons. Dantrolene is a hydantoin derivative used clinically to reduce spasticity or treat malignant hyperthermia in susceptible patients and experimental models [43,44,58], and at the cellular level, it decreases [Ca^2+^]_i_ in muscle cells [28,43,59] and neurons [26,35]. The pharmacological effects of dantrolene on [Ca^2+^]_i_ have been associated with a reduction in RyR Ca^2+^ leak [60], inhibition of Ca^2+^ release from muscle sarco-endoplasmic reticulum [61], and blocking Ca^2+^ entry into muscle cells [62]. Additionally, dantrolene prevents glutamate-induced neurotoxicity and stabilizes plasma membranes in cultured neuroblastoma cells [63,64]. Our data on dantrolene and SAR7334 further support the hypothesis that intracellular Ca^2+^ disruption serves as a necessary precursor and driver not only of aging-associated decrements in neuronal function and cognitive performance but also of the molecular mechanisms underlying the neurotoxic effects induced by isoflurane.

The neuroprotective effects of dantrolene have been observed in various animal models of neurodegenerative disorders, such as Alzheimer’s disease [58,59,60] and Huntington’s disease [65]. Furthermore, dantrolene attenuates neuronopathic phenotypes in Gaucher disease mice, suggesting its involvement in modulating Ca^2+^ signaling in neurons [66]. Although dantrolene is a promising alternative for treating neurodegenerative diseases, a significant hurdle lies in its limited ability to penetrate the blood-brain barrier (BBB) [67]. Two favorable properties of dantrolene enhance its ability to cross the BBB. First, it exhibits high lipid solubility, and second, it has a molecular weight of 314 g/mol. Drugs characterized by lipid solubility and molecular weights less than 400 g/mol are generally expected to easily traverse the BBB. However, the ability of dantrolene to penetrate the blood-brain barrier remains controversial, with evidence against [68] and for BBB passage [69]. Evidence has been published indicating that dantrolene penetrates the BBB well, (i) a tissue/plasma concentration ratio (Kp) of 6.4 in a steady state [69]. (ii) its central side effect (drowsiness and dizziness), (iii) changes in neurotransmitter levels in cerebrospinal fluid [70,71], (iv) it reduces amyloid-β load and improves learning deficits [72], (v) enhances gait walking assays in murine models of Huntington’s disease [65]. Finally, our in vivo findings demonstrate that dantrolene reduces [Ca^2+^]_i_ in cortical neurons, indicating that it crosses the BBB. Furthermore, intranasal administration of dantrolene, compared to oral delivery, has demonstrated a substantial increase in both the peak concentrations and duration of dantrolene within the brain without apparent side effects on olfaction or motor function [73].

Another draw bag of dantrolene and its clinical use is its hepatotoxicity [74]; however, a recent publication indicated that low-dose oral dantrolene (ranging from 25 to 400 mg/day) did not cause severe adverse effects and was well tolerated by most patients in a group susceptible to malignant hyperthermia with myopathic symptoms [62]. Exploring the use of dantrolene to prevent POCD represents an encouraging avenue in perioperative neuroprotection. Although its primary application remains in muscle-related conditions, its pharmacological properties (reduce neuronal [Ca^2+^]_i_) make it a plausible candidate for the treatment of cognitive deficits associated with anesthesia in older subjects.

We found a significant increase in ROS production in aged neurons (aged > middle-aged > young neurons). Elevations in ROS levels have been observed to adversely impact cellular structures and functions, potentially leading to oxidative stress, which represents a significant contributing factor to the aging process [75]. We confirmed the compelling association between [Ca^2+^]_i_ and ROS [76,77]. This intricate relationship suggests that an increase in [Ca^2+^]_i_ could uncouple mitochondria, leading to elevated ROS production, increased oxidative stress, and subsequent neuronal damage and cell death. In support of this complex interplay, we observed that treating aged mice and aged neurons with dantrolene, which is well-known to reduce [Ca^2+^]_i_ [35,44,59], also decreased ROS generation. This association seems to represent a fundamental phenomenon of aging in excitable tissues. In this context, we observed an age-dependent elevation of [Ca^2+^]_i_ and ROS production in skeletal muscle [78], whose intricate interplay became apparent when [Ca^2+^]_i_ was reduced with flufenamic acid, resulting in a notable decrease in ROS production [78]. Interestingly, isoflurane at clinical concentration exacerbated the age-related basal in ROS generation but did not affect young neurons.

Accompanying the increase in ROS, we have previously confirmed that aging increases neuronal calpain activity [26]. Calpains, a family of Ca^2+^-dependent proteases, become hyperactive in response to elevated [Ca^2+^]_i_ during pathological situations. This elevated calpain activity can degrade crucial cellular components such as ion channels, synaptic proteins, and structural elements, leading to neuronal dysfunction and, in severe cases, neuron death [48,79,80]. Furthermore, increased calpain activity has been associated with cognitive impairments in various neurodegenerative disorders [81]. Dantrolene was found to reduce calpain activity in aged neurons, possibly through its effect on lowering [Ca^2+^]_i_ [35] and/or by down-regulation of mRNA levels for m and µ-calpain [82]. The current study also found that isoflurane at clinical concentrations caused an additional increase in calpain activity in aged neurons, which could be linked to the further elevation of [Ca^2+^]_i_ caused by isoflurane. Notably, pretreatment with dantrolene prevented the increase in calpain activity produced by isoflurane. Furthermore, we found that when ROS production and calpain activation increased, LDH leakage also increased in aged > middle-aged hippocampal neurons compared to young neurons. LDH is a cytoplasmic enzyme that is used as a marker of plasma membrane integrity [83]. The shift in LDH leak observed in middle-aged and aged neurons provides the extent of plasma membrane damage, underscoring the potential impact of aging on neuronal integrity. Dantrolene mitigated the enhanced LDH leak in aged neurons, suggesting that it reduced the magnitude of plasma membrane injury, most likely through its ability to lower neuronal [Ca^2+^]_i_. Exposure to isoflurane caused an additional increase in LDH leakage in aged hippocampal neurons [84,85], which could also be prevented with dantrolene pretreatment.

Our MWM study reaffirmed the presence of age-dependent cognitive impairments without isoflurane, as we previously reported [26], and showed that isoflurane aggravated the preexistent spatial memory deficit in aged mice. Other studies have reported that isoflurane may induce neurotoxicity associated with cognitive dysfunction in humans and rodent models [86,87,88]. However, these studies have not clearly defined the molecular mechanisms leading to these changes. In the present study, cognitive impairments in aged mice were supported by (i) alteration in escape latency (aged > middle-aged >> young), indicating that aged mice took the longest time to locate the target compared to middle-aged and young mice; (ii) the time spent by mice in the target quadrant was inversely related to age (young > middle-aged > aged); (iii) the number of crossovers over the target was inversely related to age (young > middle-aged > aged). We also measured the path length due to potential and real differences in swimming speed (young > middle-aged > aged), which could interfere with escape latency values. Interestingly, we observed an age-related difference in the path length (aged > middle-aged >> young).

This indicated that the observed difference in escape latency time between young and aged mice was not due to variations in swimming speed but instead reflected age-related cognitive impairments. Exposure to isoflurane worsened escape latency, the time spent by mice in the target quadrant, and the number of crossovers over the target with age (aged > middle-aged), with no effect on young mice. Importantly, exposure to isoflurane did not alter swimming speed in any age group. This observation suggests that motor deficits in aged mice were not the primary contributors to spatial memory impairments observed after recovery from isoflurane, emphasizing the cognitive impact of isoflurane on aged mice. The neuroprotective efficacy of dantrolene has been substantiated in various models of neurodegenerative disorders [65,89]. Our previous research demonstrated that in a normal aging model, pretreatment with dantrolene exhibits a neuroprotective influence on aged neurons, thus ameliorating cognitive deficits associated with aging [26]. In the current study, evidence indicates that reducing abnormal basal [Ca^2+^]_i_ with dantrolene prevents intracellular Ca^2+^ overload induced by isoflurane and the worsening of cognitive function in aged mice.

## 5. Study Limitations

Although our study presents valuable information about the mechanism involved in causing POCD, it is essential to acknowledge several limitations: (i) Our investigation focused solely on exploring the hypothesis that intracellular Ca^2+^ dysregulation is a mechanism for post-anesthesia cognitive decline. (ii) We used MWM to assess spatial learning, a test known to potentially induce anxiety in mice, as evidenced by increased corticosterone levels in their plasma [90]. We did not measure corticosterone levels in this study; therefore, we did not evaluate the potential impact of the stress response in different age groups. (iii) The visual acuity of the aged mice was not investigated. Therefore, we cannot exclude the potential influence of cataracts on the performance of aged mice in MWM, but this possibility seems unlikely because performance could be improved with dantrolene, which has not been shown to improve eyesight. (iv) Our study did not explore the role of gender on the effects of isoflurane and dantrolene on spatial learning and memory in aged mice.

## 6. Conclusions

In this study, we reaffirmed our previous findings that [Ca^2+^]_i_ is elevated in middle-aged and aged cortical neurons (in vivo) and pyramidal hippocampal neurons (in vitro). We also demonstrated a clear association between this increase in [Ca^2+^]_i_ in aging neurons with increased ROS production, LDH leak, and calpain activity. To our knowledge, no previous studies have shown a direct link between isoflurane, dysregulation of neuronal [Ca^2+^]_i_, the development of POCD in aged mice, and the neuroprotective effect of dantrolene. The findings underscore a connection between isoflurane-induced disruptions in neuronal [Ca^2+^]_i_ and cognitive decline in aging.

The ability of dantrolene to prevent isoflurane-induced neuronal Ca^2+^ overload and mitigate preexisting cognitive decline in aged mice positions this muscle relaxant as a promising therapeutic option for preserving cognitive function in elderly individuals undergoing general anesthesia. As accumulating evidence supports its efficacy, the consideration of incorporating dantrolene into perioperative strategies becomes increasingly viable. This is particularly noteworthy as new administration routes have been identified, facilitating higher concentrations in the brain with minimal side effects.

## Data Availability

The datasets generated and analyzed during the current study are available from the corresponding author upon request.

## Supplementary Materials

The following supporting information can be downloaded at: https://www.mdpi.com/article/10.3390/cells13030264/s1, Figure S1: SAR-7334 reduces abnormal resting [Ca^2+^]_i_ and inhibits isoflurane-induced further elevation in aged hippocampal neurons (in vitro).
