# Supplementary material for: Post-Anesthesia Cognitive Dysfunction in Mice Is Associated with an Age-Related Increase in Neuronal Intracellular [Ca2+]—Neuroprotective Effect of Reducing Intracellular [Ca2+]: In Vivo and In Vitro Studies
Source: Cells. 2024 Jan 31;13(3):264. doi: 10.3390/cells13030264 (PMC10854970; doi:10.3390/cells13030264)
Supplement: Supplementary file 1 [file cells-13-00264-s001.zip › cells-2767335-supplementary.pdf]

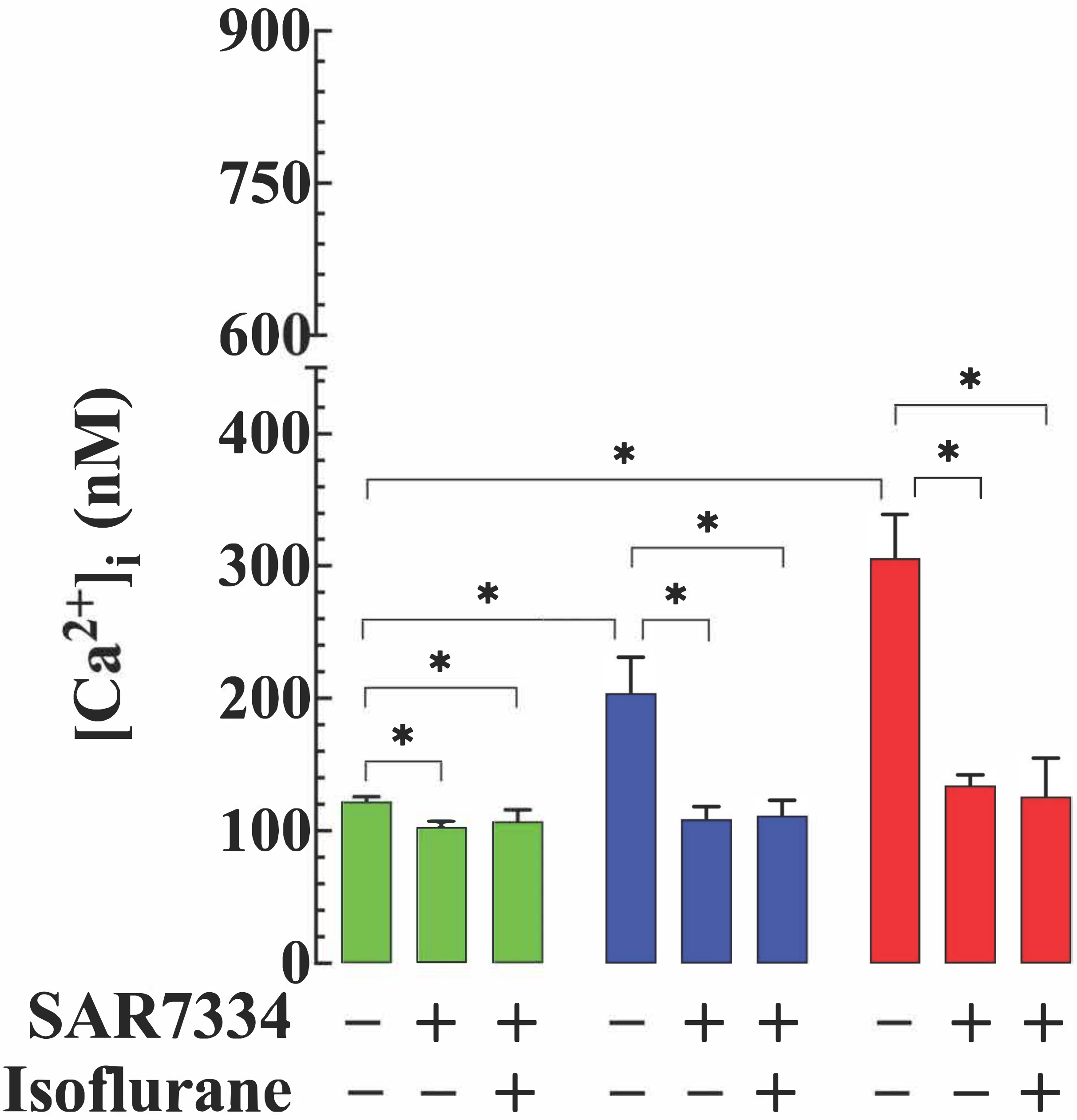

Supplemental Figure S1. SAR-7334 reduces abnormal resting $[Ca^{2+}]_i$ and inhibits isoflurane-induced further elevation in aged hippocampal neurons. A. The average $[Ca^{2+}]_i$ in hippocampal middle-aged neurons was 214±23 nM (n_{cells} =18, p<0.05 compared to young neurons) and in aged neurons 311±39 nM (n_{cells} =12, p<0.05 compared to young neurons), while that in young neurons was 121±3 nM (n_{cells} =13). SAR-7334 (1 μM) reduced $[Ca^{2+}]_i$ in middle-aged neurons to 98±4 nM (n_{cells} =13, p< 0.05 compared to untreated age match neurons), in aged neurons to 101±8 nM (n_{cells} =10, p< 0.05 compared to untreated age match neurons) and in young neurons to 94±6 nM (n_{cells} =13, p< 0.05 compared to untreated age match neurons). Pretreatment with SAR-7334 inhibited isoflurane- induced increase $[Ca^{2+}]_i$ in middle-aged mice and aged neurons. n_{mice}= 5/age group-experimental condition. Values are expressed as mean ± S.D. *denotes p<0.05.
